# MDAD: A Special Resource for Microbe-Drug Associations

**DOI:** 10.3389/fcimb.2018.00424

**Published:** 2018-12-07

**Authors:** Ya-Zhou Sun, De-Hong Zhang, Shu-Bin Cai, Zhong Ming, Jian-Qiang Li, Xing Chen

**Affiliations:** ^1^Department of Computer Science and Technology, College of Computer Science and Software Engineering, Shenzhen University, Shenzhen, China; ^2^School of Information and Control Engineering, China University of Mining and Technology, Xuzhou, China

**Keywords:** database, drug, microbe, drug target, drug discovery

## Abstract

The human-associated microbiota is diverse and complex. It takes an essential role in human health and behavior and is closely related to the occurrence and development of disease. Although the diversity and distribution of microbial communities have been widely studied, little is known about the function and dynamics of microbes in the human body or the complex mechanisms of interaction between them and drugs, which are important for drug discovery and design. A high-quality comprehensive microbe and drug association database will be extremely beneficial to explore the relationship between them. In this article, we developed the Microbe-Drug Association Database (MDAD), a collection of clinically or experimentally supported associations between microbes and drugs, collecting 5,055 entries that include 1,388 drugs and 180 microbes from multiple drug databases and related publications. Moreover, we provided detailed annotations for each record, including the molecular form of drugs or hyperlinks from DrugBank, microbe target information from Uniprot and the original reference links. We hope MDAD will be a useful resource for deeper understanding of microbe and drug interactions and will also be beneficial to drug design, disease therapy and human health.

## Introduction

Microorganisms exist everywhere, from soils to oceans, air, plants, animals and humans. It is well known that bacterial or viral infection can result in severe infectious diseases. Antibiotics have saved millions of patients in the more than 70 years since penicillin was discovered in the 1940s. Today, several classes of antimicrobial agents are available (Plotkin and Plotkin, 2011). Their mechanisms of action include inhibiting bacterial protein biosynthesis (gentamicin, doxycycline), interfering with DNA (ciprofloxacin, enoxacin), inhibiting cell wall synthesis (ampicillin, cefadroxil), interfering with the cytoplasmic membrane (daptomycin, colistin) and so on (Labro, 2012). However, a major concern for antibiotic resistance is emerging. In recent decades, drug-resistant bacteria have spread at alarming rates and become a serious public health threat. Meanwhile, the rate of new antibiotic discovery decreased. In the past 30 years, only two new classes of antibiotics have been developed, of which there were no new classes of antibiotics for Gram-negative infections (Trusts, 2014). Drug resistance emerged even before the drug was approved by Food and Drug Administration (FDA) (Bax et al., 1998). In fact, antibiotic resistance has led to many standard antibiotics in general losing their effectiveness. It is becoming difficult to treat infectious diseases that were once easily curable with antibiotics. The number of patients suffering severe illness or dying has increased. According to a report from the United Kingdom government, if there are no new strategies for antimicrobial discovery, it is predicted that antibiotic-resistant infections will result in 10 million deaths worldwide every year by 2050 (O'neill, 2014).

To combat antibiotic resistance, new strategies and approaches are needed. A new strategy to overcome drug resistance is drug combination research (Zimmermann et al., 2007). The first use of combinatorial drugs was explored in tuberculosis with antibacterial drugs (1948). Based on this successful application, combinatorial drug therapy is becoming widely used in the treatment of HIV infections and cancer chemotherapy (Vandamme et al., 1998). Another strategy is drug repositioning, which means exploring new or unexpected therapeutic effects of old drugs. For both combinatorial drug treatment and drug repositioning, accurate and effective identification of microbe-drug interactions is the first step (Chen et al., 2016b).

Past microbial studies have shown the potential for new discoveries in various areas. Studies of the human microbiome have also indicated its important role in human health and behavior. The microbial communities in the human body exist in the skin, mouth, gastrointestinal tract, vagina and other tissues. These microbes are important for the homeostasis of the organism's internal environment (Elrakaiby et al., 2014). The imbalance or dysbiosis in these populations is also related to noninfectious diseases, including diabetes, obesity, rheumatoid arthritis, cancer and depression (Lynch and Pedersen, 2016). For instance, the disorder of human microbiota can inactivate pharmaceuticals (Haiser et al., 2013) or increase the risk of cardiovascular diseases (Koeth et al., 2013). Vaginal dysbiosis can increase the risk of preterm birth, HIV transmission and other pelvic inflammatory diseases (van de Wijgert and Jespers, 2017). Some oral diseases (e.g., caries) and skin diseases (e.g., dermatitis) are also significantly impacted by microbes (Avila et al., 2009; Kong and Segre, 2012). The human body is a complex host of many systems. On a broad scale, researchers need to understand how the microbiome functions. This knowledge will be essential to revealing the impact of microbes on human health. In fact, efforts to explore the key role of microbiota in the human body have become the frontier of biological and medical research. The Human Microbiome Project (HMP), launched in 2007 (Turnbaugh et al., 2007), is a project to evaluate the diversity and function of the human microbiome regarding health and disease. It has already published a full description of the microbiome in five human body tissues, including the skin, gut, nares, vagina, and oral cavity (Aagaard et al., 2013). The National Microbiome Initiative (NMI) also aims to explore the impact of microbial systems on healthcare and other areas. The achievements in these projects indicate the great potential for applications of intraindividual microbiomes in personalized therapy (Wang et al., 2017). For example, the gut microbiota is highly sensitive to microenvironmental factors such as nutritional supplement and diet. These factors may have impacts on drugs such as antibiotics or chemotherapeutic agents, especially when the balance of gut microbiota is disturbed. Thus, it seems reasonable to add microbiome assessment to cancer chemotherapy protocols, which may increase the efficacy and safety of the treatment. Deep understanding of the relationship between microbes and clinical chemicals is essential for the development of personalized medicine. Therefore, there is an urgent need for special databases and powerful approaches that can reveal the complex microbe-drug associations.

Nowadays, there are a number of drug-related databases, including DrugBank (Law et al., 2014), the Therapeutic Target Database (TTD) (Qin et al., 2014), SuperTarget (Hecker et al., 2012) and so on. DrugBank is a comprehensive database that combines quantitative drug data and detailed target information (Law et al., 2014). TTD provides information about known therapeutic targets from literature as well as related disease conditions, corresponding drugs and pathway information (Qin et al., 2014). SuperTarget is also an extensive database that integrates drug-target interactions from different resources (Hecker et al., 2012). In addition, DCDB is a database that provides information on drug combinations from clinical studies in the FDA's Electronic Orange Book (Liu et al., 2014). Recently, several databases related to microbes and drugs have been developed. The Comprehensive Antibiotic Resistance Database (CARD) is a resource focusing on antibiotic resistance (Jia et al., 2017). ResFinder is a web server using whole-genome sequencing (WGS) data to identify antimicrobial resistance genes by BLAST (Zankari et al., 2012). The aBiofilm is a resource containing experimentally validated anti-biofilm agents (Rajput et al., 2018). The PharmacoMicrobiomics Portal is a resource for drug-microbiome interaction gained from HMP, but it contains a very limited number of records (Rizkallah et al., 2012). To our knowledge, no special platform provides concise yet comprehensive associations between microbes and drugs, especially non-antibiotic drugs. A high-quality database related to the associations between microbes and drugs is essential to understand the mechanisms of microbes in clinical treatment, which is important for the development of therapeutics, drug discovery, combinations and repositioning. In this article, we developed a comprehensive database, the Microbe-Drug Association Database (MDAD), which integrates a variety of data from different resources. The goal of MDAD is to help researchers and clinicians to investigate the relationship between microbes and drugs, and finally to facilitate antimicrobial drug discovery and the application of microbiomes in personalized medicine.

## Materials and Methods

### Data Collection and Compilation

**Figure 1:**
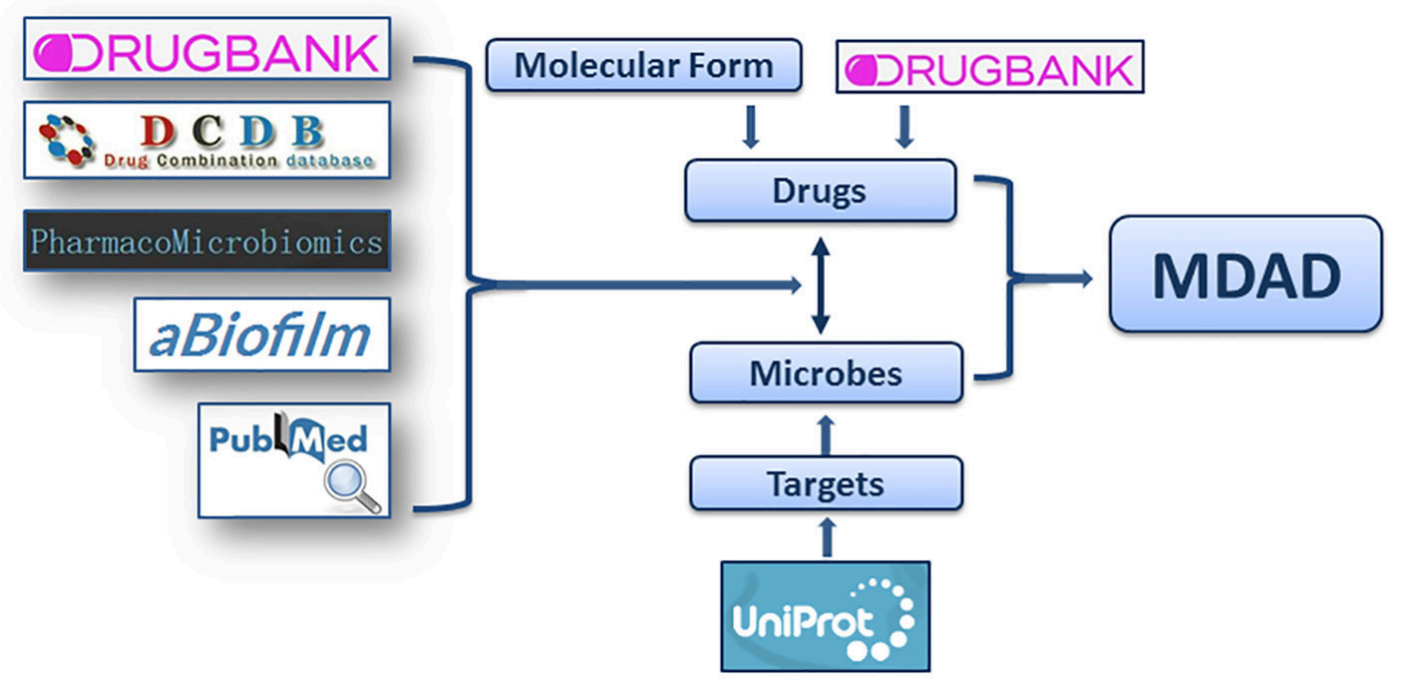
The flowchart of MDAD construction. The flowchart shows the process of data processing and information integration.

Considering that the study about microbe-related drugs has been going on for decades and many antimicrobial drugs have been reported, we firstly collected the microbe-drug associations from present drug-target databases. We browsed almost all drug databases, including DrugBank (Law et al., 2014), TTD (Qin et al., 2014), SuperTarget (Hecker et al., 2012), MATADOR (Gunther et al., 2008), STITCH (Kuhn et al., 2014), TDR targets (Magarinos et al., 2012), PDTD (Gao et al., 2008), ChEMBL (Gaulton et al., 2012), aBiofilm (Rajput et al., 2018), and Integrity (Emig et al., 2013). After removing duplicate terms, we collected more than 4,000 entries mainly from DrugBank (Law et al., 2014), DCDB (Liu et al., 2014), PharmacoMicrobiomics (Rizkallah et al., 2012), and aBiofilm (Rajput et al., 2018). All the records from these resources should contain clear information of drugs, microbes and the references confirming their associations. Then, considering that the updates of previous databases may be not current, we also collected the microbe-drug associations from the latest references of the last 2 years. We performed an extensive literature search in PubMed using a list of keywords, including “microbe,” “microbial,” “microorganism,” and “drug,” “drug targets,” “chemical” etc. The search resulted in about 1,500 articles that from the last 2 years. We filtered about 500 articles that may contain microbe-drug association data by excluding non-potential articles and reviews. Then we manually retrieved microbe-drug associations by reading abstracts or full texts from the 500 filtered articles. Finally, about 1,000 entries from more than 100 recently published references were collected (Figure 1). The experimentally supported associations were demonstrated by lab experiments reported in publications. The clinically supported microbe-drug associations were FDA-approved or confirmed to be effective in clinical trials. We only chose entries with clear experimental or clinical evidences. Then the names of microbes and drugs were further standardized. To aid users in getting more information, we provided annotations or hyperlinks for drugs and microbes. We also provided the PubMed ID and hyperlinks of the original articles for each association (Wheeler et al., 2004).

### Database Design and Implementation

The MDAD database was organized using the MySQL database management system (a kind of Relational Database Management System) on an X86–64 server with Windows Server 2012 OS. The user interface was presented by Hyper Text Markup Language (HTML), Cascading Style Sheets (CSS), JavaScript, jQuery, and Bootstrap frameworks. The functions for database query, corresponding browser and submission were implemented by PHP: Hypertext Preprocessor (PHP), Structured Query Language (SQL), and Apache2 (Gabarro, 2007). MDAD is available at http://chengroup.cumt.edu.cn/MDAD and all the corresponding codes are publicly available (https://github.com/Sun-Yazhou/MDAD). We have tested the web server with most common browsers, including Google Chrome, Internet Explorer and Mozilla Firefox. The response time for the first page request to the MDAD website is usually within seconds. Normally, the database query, browser and submission could be completed in < 1 s. We used Pingdom tools (https://tools.pingdom.com) to conduct the website test. The performance grade of MDAD was 81. The number of requests was 15, while the load time was 4.84 s.

## Results

### Data Statistics and Content

**Figure 2:**
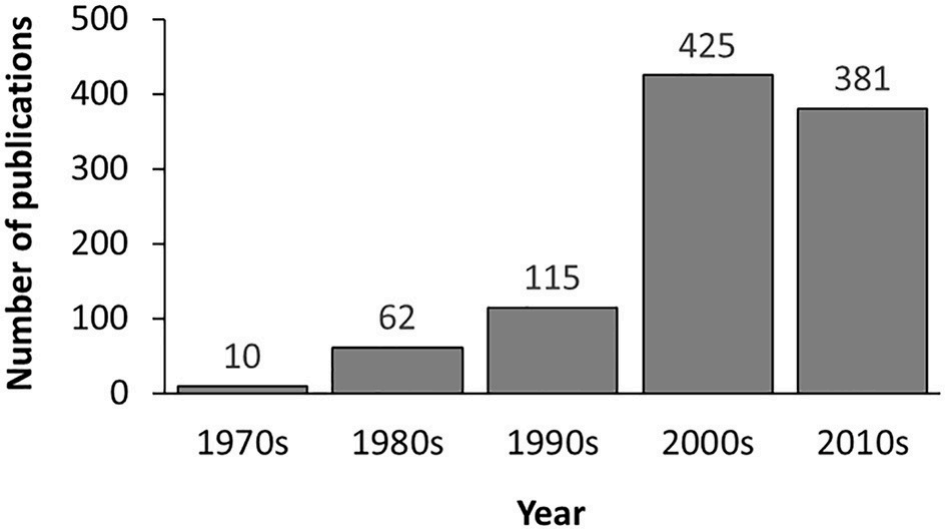
The distribution of references included in MDAD by time of publication.

**Figure 3:**
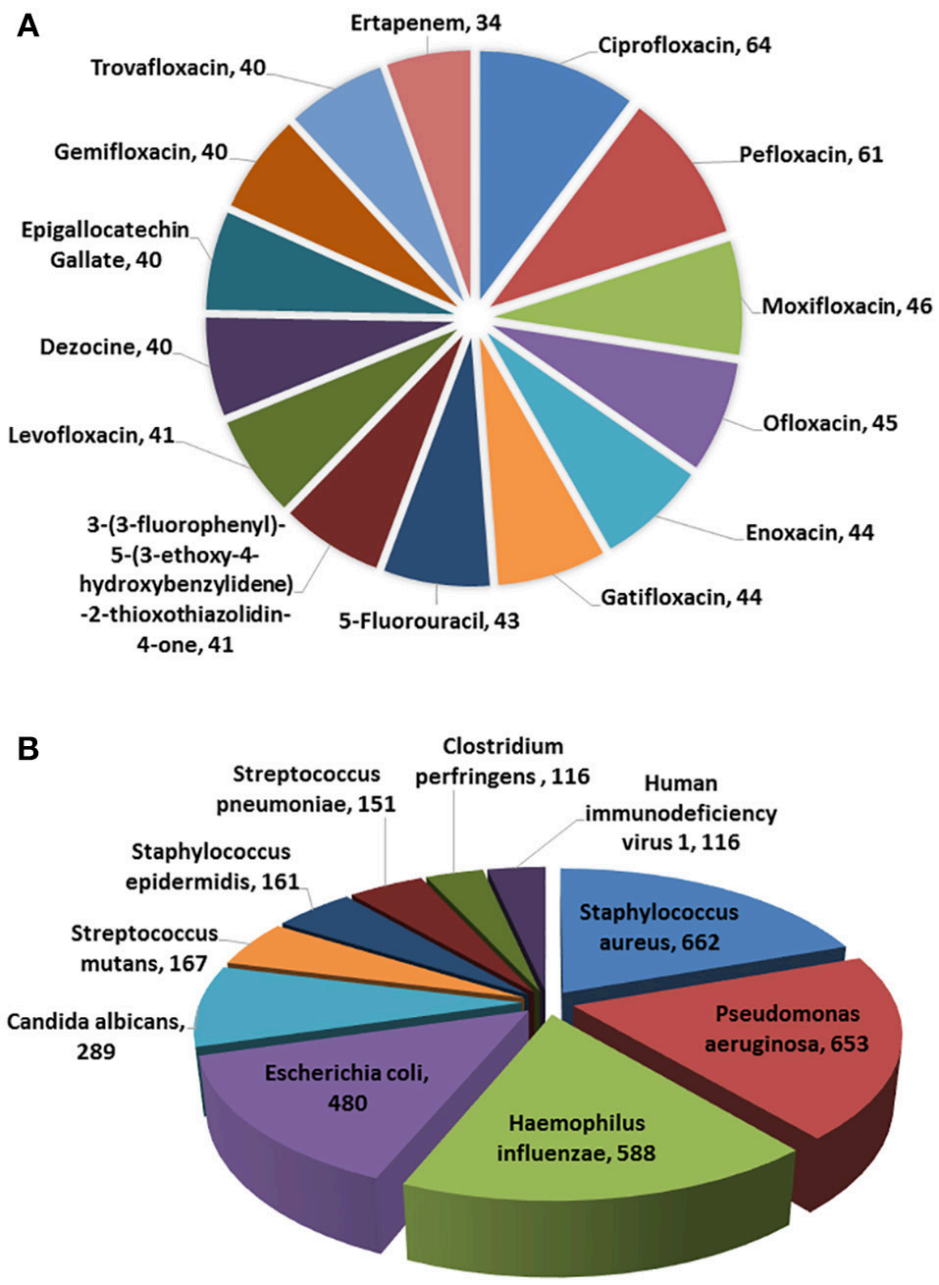
Diagrammatic representation of **(A)** topmost drugs in MDAD, **(B)** topmost microorganisms in MDAD.

In the current version, MDAD collects 5,055 entries, including 1,388 drugs, 180 microbes and 824 different strains (not including the microbes without specified strains) related to 993 references. All the references were from the 1970s to the time of writing (Figure 2). The number of references significantly increased in the 2000s, which might be due to the increasing emergence of antibiotic resistance and new discoveries in microbiome projects. Among the topmost drugs, ciprofloxacin has 64 entries, followed by pefloxacin, moxifloxacin, ofloxacin, Enoxacin, gatifloxacin and 5-fluorouracil, with 61, 46, 45, 44, 44, and 43 records (Figure 3A). Notably, apart from the drugs belonging to classical antimicrobial agents, there were also drugs related to non-infectious diseases, such as 5-fluorouracial, which is widely used in cancer treatment. The database has the most drug records related to *Staphylococcus aureus*, with 662 hits, followed by *Pseudomonas aeruginosa, Haemophilus influenza, Escherichia coli, Candida albicans, Streptococcus mutans* with 653, 588, 480, 289, 167 hits (Figure 3B). All of these microbes are widespread in environment, human body and other organisms, which are involved in various diseases. Every entry in MDAD contains three major fields, i.e., drug name, microbe name and the PubMed ID reference (hyperlinked to PubMed). We further annotated the drug with molecular form or links to DrugBank (Law et al., 2014). Other fields include strain and target of microbes. The types of these targets are usually proteins or RNAs. They are the direct targets in the microorganisms for drugs. Specifically, we provided the links to Uniprot for the targeting proteins (Consortium, 2015).

### The MDAD Web Interface Organization and Functionality

**Figure 4:**
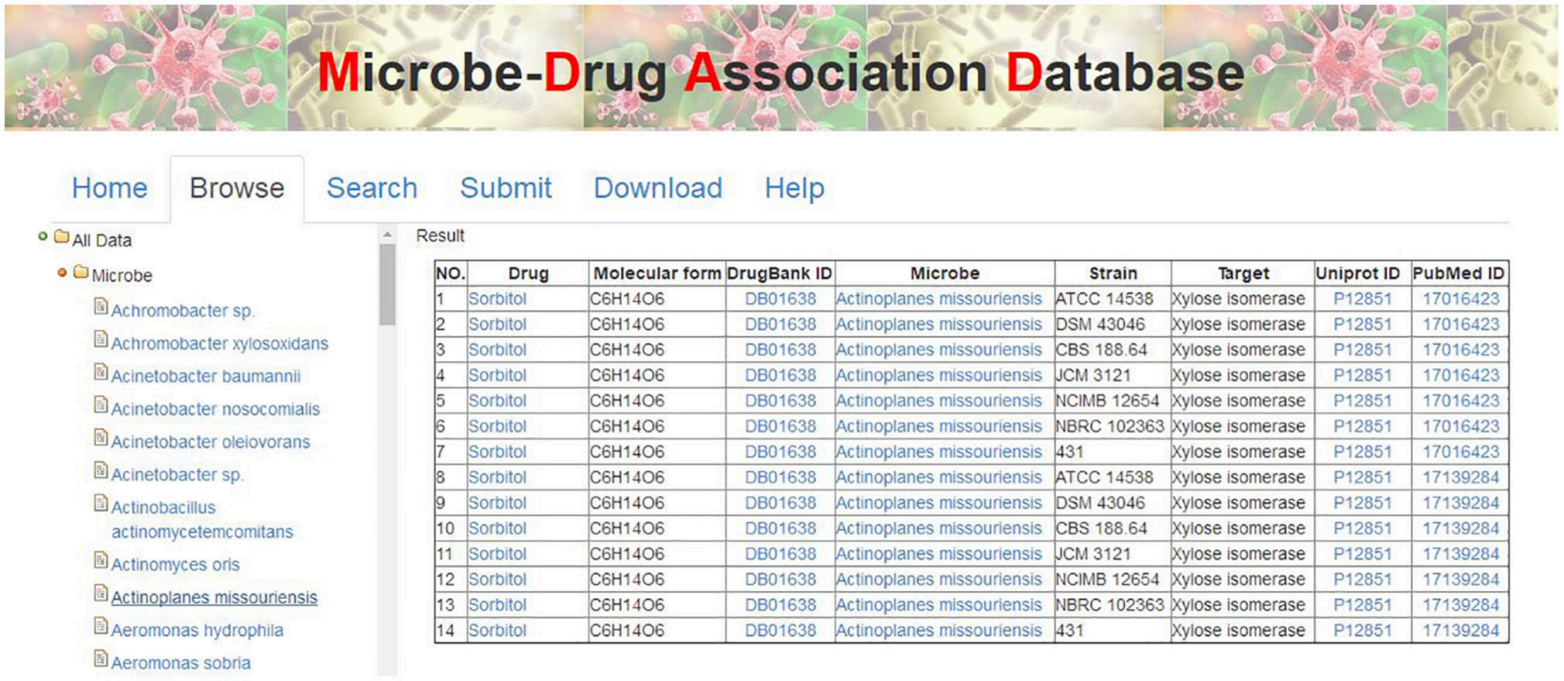
The MDAD user interface showing the browse page.

There are various ways to get the data in MDAD, including table browser, entry search and downloading. First, users can browse the MDAD to explore and scan the overall entries by microbe or drug names. Users can click the menu “Browse” and select the microbe or drug that they are interested in. MDAD will return the corresponding list of entries in the right panel. For example, users can click “Microbe” first and select “*Actinoplanes missouriensis*.” The result will be shown in the right panel (Figure 4). Additionally, through the internal links in MDAD, users can easily explore the associations between drugs and microbes. For example, in the previous selected entries of *Actinoplanes missouriensis*, if users want to explore the effects of the given drug “Sorbitol” on other microbes, they can click it and get an output of all the entries related to “Sorbitol.” They will find that “Sorbitol” also has effects on *Arthrobacter sp., Escherichia coli* and *Streptomyces rubiginosus*. Second, we have also provided a “search” tool to meet the need of information according to the user's own requirements. Users can enter the query by drug name, microbe name or PubMed ID in the “Search” page. Moreover, all data regarding drug–microbe associations in MDAD, including all drug names, microbe names, strain names, target names, and PubMed ID, can be downloaded.

Apart from data retrieval from MDAD, users can submit their own data into the database. They are encouraged to search MDAD to check whether the data have already been deposited into the database. If not, they can upload the entries with drug name, microbe name, PubMed ID and other related information. The novel data will be forwarded to the MDAD developers via email. After passing a manual check and confirmation, the data will become available. To get a detailed tutorial for the usage of MDAD, the users can scan the “Help” page.

### The MDAD Used in Microbe-Drug Association Study

**Figure 5:**
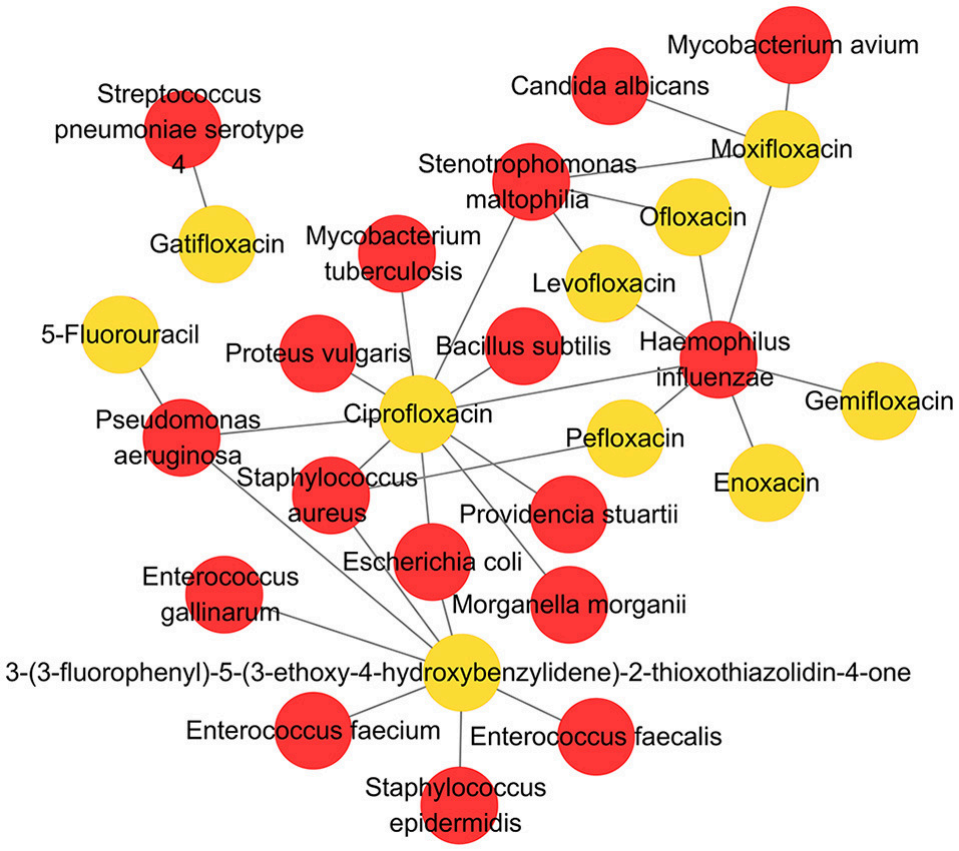
The network map of associations between top 10 drugs and related microbes.

As a high-quality database, MDAD is useful for the research of microbe-related drug discovery. It provides verified associations between microbes and drugs, which are essential for the detection of detailed mechanisms and the potential relationship between them. For example, cefotaxime can kill *Acinetobacter baumannii* by targeting beta-lactamase. In MDAD, we can find many other drugs that target beta-lactamase, including aztreonam, cloxacillin, imipenem and so on. Although there may not be direct associations between these drugs and *Acinetobacter baumannii* at present, we can consider their efficiency against *Acinetobacter baumannii* with the same target. Furthermore, research can be designed and those confirmed drugs might be added to the synergistic drug combination strategy. Additionally, as shown in Figure 5, we used Cytoscape to make a network map representing the associations among the top 10 drugs and their related microbes. The map displays some important microorganisms such as *Escherichia coli, Pseudomonas aeruginosa, Staphylococcus aureus, Haemophilus influenza*, and their corresponding antibiotic agents, including ciprofloxacin and pefloxacin. In particular, the map indicates the associations between these microorganisms and non-antibiotic drugs such as 5-fluorouracil and 3-(3-fluorophenyl)-5-(3-ethoxy-4-hydroxybenzylidene)-2-thioxothiazolidin-4-one. The former is widely used in cancer treatment while the latter is still not fully studied (Figure 5). It suggests the great potential of these microorganisms in medical precision and drug repositioning. Meanwhile, based on these verified network data, scientists can develop computational tools to explore the potential associations that are not shown in the map at present. Therefore, MDAD will greatly benefit the prediction of microbe-drug associations, which could facilitate further experimental drug screening. In the future, the MDAD will be updated continually and several computational tools will be integrated for the prediction of novel microbe-drug associations.

## Discussion

Microbes are closely related to human healthcare. Antimicrobial drugs have saved millions of lives in the last century. New applications of microbes in personalized medicine and clinical drug treatment are becoming increasingly attractive. These achievements represent the application of two major types of microbe-drug associations: microbes as drug targets and pharmacomicrobiomic interactions. Although the microorganisms have been studied for many years, antibiotic resistance and new discoveries in HMP bring forward new challenges and higher requests in this field. There is an urgent need to integrate platforms to obtain comprehensive and high-quality data about the relationships between microbes and drugs easily.

In this article, we presented the MDAD, which is a special database focusing on microbe-drug associations. It integrated various types of data from different resources with both major types of microbe-drug associations. While keeping the comprehensiveness of the data, we organized the web interface to be concise and user-friendly. The MDAD will facilitate microbe and drug research by providing users with direct and comprehensive associations between microbes and drugs that are clinically or experimentally confirmed. Based on these high-quality data, researchers may understand more deeply microbe-related drug advancements. It will significantly improve the accuracy and efficiency of synergistic drug combinations and drug repositioning as well as contribute to solving actual biological problems.

The current MDAD represents the first step of our whole microbe-drug research project. It still has limits in data analysis. The form of displaying records is simple. Besides, the current version focuses on the one-to-one relationship between a specific microbe and drugs but does not include microbiome variation data. In the future, we plan to update MDAD continually with data from newly published references, submitted entries via users and updated data in other drug-target databases. Meanwhile, we will develop and incorporate new analysis and visualization tools in MDAD. For example, we will develop computational methods to predict novel microbe-drug associations base on the previous studies in our group (Chen et al., 2012, 2016a, 2018) and integrate them in MDAD. With more abundant datasets and methods of mathematical analysis suitable for complex datasets, we will make further efforts to integrate the gene expression analysis data of entire microbial communities related to drugs in the disciplines of metagenomics. It will provide more help while integrating these data and tools in the future. We believe that MDAD will help researchers to understand the relationship between microbes and drugs and further intensify the study on drug discovery and disease therapy.

## Database Update

An important aspect of any database is to keep it up to date by adding new data. We will constantly add information about new experimentally or clinically supported disease-related microbe-drug association data.

## Accessibility and Data Download

MDAD is publicly accessible at http://chengroup.cumt.edu.cn/MDAD with all major browsers supported.

## Author Contributions

Y-ZS prepared the manuscript, collected and organized the data. D-HZ and Y-ZS developed the web interface. XC conceived the project, analyzed the results and revised the paper. J-QL conceived the project and analyzed the results. ZM conceived the project and revised the paper. S-BC analyzed the results. All authors read and approved the final manuscript.

### Conflict of Interest Statement

The authors declare that the research was conducted in the absence of any commercial or financial relationships that could be construed as a potential conflict of interest.
